# Building research capacity through “Planning for Success”

**DOI:** 10.1371/journal.pntd.0007426

**Published:** 2019-08-01

**Authors:** Ligia Gómez, Andrés Jaramillo, Beatrice Halpaap, Pascal Launois, Luis Gabriel Cuervo, Nancy Gore Saravia

**Affiliations:** 1 Centro Internacional de Entrenamiento e Investigaciones Médicas, Cali, Colombia; 2 Universidad Icesi, Cali, Colombia; 3 UNICEF/UNDP/World Bank/WHO Special Programme for Research and Training in Tropical Diseases, World Health Organization, Geneva, Switzerland; 4 Health Services and Access Unit, Department of Health Systems and Services of the Pan-American Health Organization/World Health Organization, Washington, DC, United States of America; Saudi Ministry of Health, SAUDI ARABIA

## Introduction

Strengthening health research capacity in low- and middle-income countries (LMICs) has been identified as a driver of development and requirement for efficient investment of limited resources. Among the unique challenges that LMICs face when undertaking research for health are a persistent shortage of experienced researchers and competent interdisciplinary research teams, limited research career opportunities [1, 2, 3], intense competition for scarce resources targeting LMIC priorities [3, 4, 5], and an urgent need for translating research results into policy and practice [3, 4, 5]. One common denominator of these challenges is the need for sustainable capacity in planning, monitoring, and evaluating health research projects [1, 3, 6, 7]. How to effectively build these management skills among health researchers and their project teams is a persistent concern [8,9] because such capacities are generally not included in academic degree programs [9], and investigators already immersed in teaching, clinical, and research responsibilities have limited time to pursue further training [9].

Since 2007, TDR, the Special Program for Research and Training in Tropical Diseases cosponsored by United Nations Development Programme (UNDP), UNICEF, the World Bank, and WHO; institutions serving as Regional Training Centers (RTCs); and diverse government and regional partner entities synergized efforts towards building project management capacity for health research, especially for neglected tropical diseases (NTDs) [10]. Together, they have led the regional dissemination of the “Planning for Success” initiative based on two training courses: Skill-Building for effective planning, implementation, monitoring, and evaluation of health research projects and a Train-the-Trainer (TTT) course to ensure availability of local trainers.

Our goal in this Viewpoint is to consider unique features of the “Planning for Success” approach to project management training and its regional dissemination model and provide insight into their applicability to other needs for health-research-capacity–strengthening.

## Sustainable implementation of effective project planning and evaluation in health research

**Fig 1:**
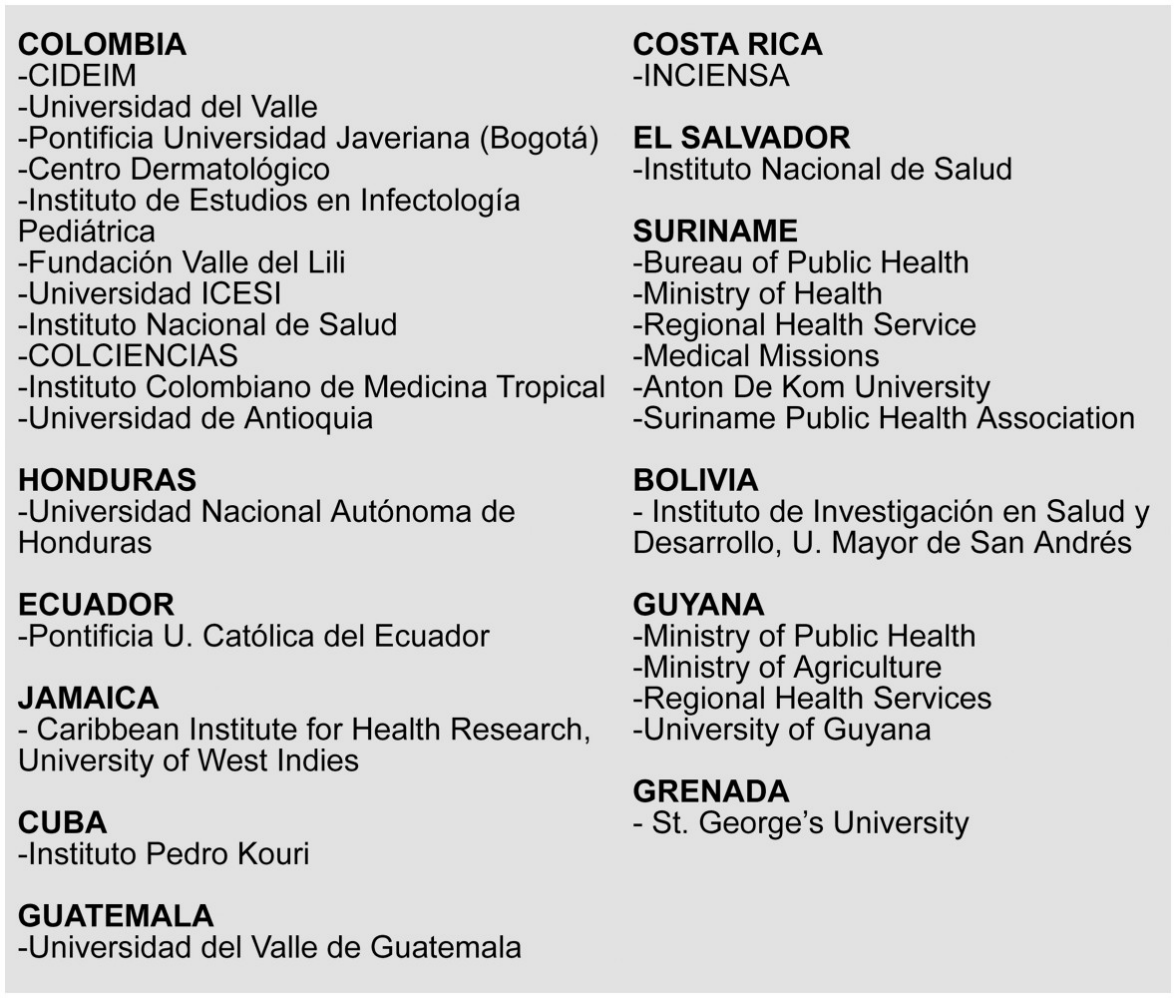
Institutions involved in the dissemination of EPPE in health research in the Latin America and Caribbean region. CIDEIM, Centro Internacional de Entrenamiento e Investigaciones Médicas; COLCIENCIAS, Administrative Department of Science, Technology and Innovation of Colombia; EPPE, Effective Project Planning and Evaluation; Universidad ICESI, Instituto Colombiano de Estudios Superiores de Incolda; INCIENSA, Instituto Costarricense de Investigación y Enseñanza en Nutrición y Salud.

**Fig 2:**
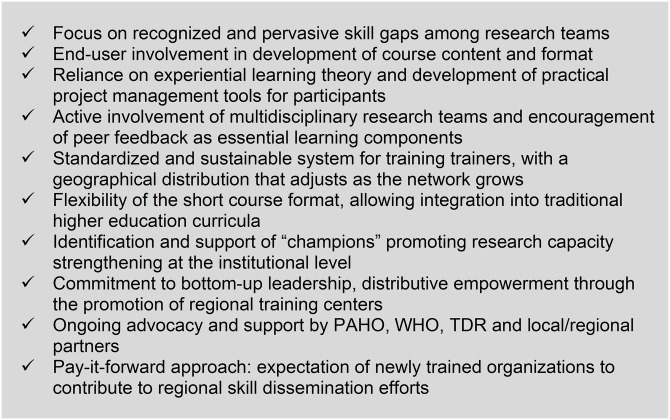
Keys to success of the “Planning for Success” initiative (identified by RTC and network institution trainers based on postcourse and program assessment). PAHO, Pan American Health Organization; RTC, Regional Training Center; TDR, XXX.

The cornerstone of the “Planning for Success” initiative is the Skill-Building course on Effective Project Planning and Evaluation (EPPE) that takes participants through each of the phases of project management, i.e., project planning, implementing, monitoring, and evaluating. Embedding of training in project planning and evaluation within an organization lays the cornerstone for institutionalization of this capacity as an organizational practice. Centro Internacional de Entrenamiento e Investigaciones Médicas (CIDEIM) was initially supported for one year by TDR as a reference center for EPPE training in 2007 [10] in order to implement local and regional dissemination of this training. Since 2009, as an RTC for good research practices, CIDEIM—together with collaborating Latin American and Caribbean network institutions—has infused project management principles into their organizational culture. Institutional “champion” facilitators/trainers conduct regular internal courses, harmonizing project planning and evaluation tools with existing institutional and external systems of research administration and promoting the organizational value of this expertise. The uptake of this practical planning strategy is evidenced by the global and regional dissemination of the course (Fig 1) and its incorporation into diverse undergraduate, postgraduate, and continuing education programs (Table 1). In the following section, we describe key characteristics of EPPE training identified by trainers and participants as contributory to its relevance, engaging dynamic, and translation to practice (Fig 2).

**Table 1:** Institutionalization of EPPE training.

**Incorporation of EPPE into Undergraduate and Postgraduate University Degree Programs**		
**Location**	**Institution**	**Program/course name**
Tegucigalpa, Honduras	Universidad Nacional Autónoma de Honduras	Master’s in public health program/project planning and evaluation module
Quito, Ecuador	Pontificia Universidad Católica de Ecuador	School of Biological Sciences, College of Exact and Natural Sciences/planning and conducting the undergraduate thesis project Master’s in Biology of Infectious Diseases program/project planning
Guatemala City, Guatemala	Universidad del Valle de Guatemala	Master’s in epidemiology/research methods Undergraduate programs in science and humanities/research methods
Kingston, Jamaica	University of West Indies at Mona	Master’s of science in epidemiology and forensics/research methods
Cali, Colombia	Universidad del Valle	Doctoral program in health, Faculty of Health/project formulation and evaluation
Bogotá, Colombia	Pontificia Universidad Javeriana de Bogotá	Graduate program (MSc and PhD) in biological sciences/EPPE in biomedical research
Cali, Colombia	Universidad Icesi	Undergraduate programs in natural sciences (biology, chemistry, pharmaceutical chemistry/undergraduate thesis project development)
**Incorporation of EPPE into Continuing Education Programs**		
Cali, Colombia	CIDEIM	Promotion and development of research capacity unit/effective project planning in health research
La Habana, Cuba	Instituto de Medicina Tropical “Pedro Kouri”	Project planning and evaluation for project managers
Medellin, Colombia	Universidad de Antioquia	Research Center of the National Faculty of Public Health/EPPE in biomedical research
Tegucigalpa, Honduras	Universidad Nacional Autónoma de Honduras	Scientific Research Unit, Faculty of Medical Sciences/Research Methodology course

**Abbreviations**: CIDEIM, Centro Internacional de Entrenamiento e Investigaciones Médicas; EPPE, Effective Project Planning and Evaluation.

### Responding to a felt need and involving end-users

In 2002, TDR identified the need for ensuring efficient, timely, and cost-effective conduct of projects and management of grants funded through its Multilateral Initiative on Malaria (MIM), which aimed to “maximize the impact of scientific research against malaria in Africa, through promotion of capacity building activities and facilitating global collaboration and coordination” [11]. The EPPE Skill-Building course was crafted specifically to address this multifaceted need. Notably, intended beneficiaries of the imparted skills contributed to the development of course content and format, thereby enhancing its relevance and applicability.

Several models of project management training existed when the EPPE course was developed [12]. However, many health researchers considered project management to be outside their responsibilities or simply inaccessible [12].

The Skill-Building course developers intentionally tailored the course to the multidisciplinary backgrounds and roles of the diverse professionals involved in health research. Terms, concepts, and learning methods are conveyed through a specific case study example and immediate application to participants’ own research projects. Project management concepts following a results-based management approach including alignment of objectives, definition of roles, coordination of activities and timelines, and effective mechanisms of communication are presented in practical terms, emphasizing the value of the appropriation and application of project planning and evaluation by research teams.

### Step-by-step learning

Rooted in David Kolb’s experiential learning theory [13], course content is presented in a step-by-step process, with each module building upon the previous one. This progressive and experience-based approach allows participants to consider theory, reflect through illustrative case studies, and then cement their learning by applying the new concepts to planning their own projects. Participants reach the end of the course with a realistic and achievable plan and a set of tools for monitoring the implementation of their projects. Upon completion of each course, four aspects of the training have been systematically evaluated: content, facilitators, organization, and applicability of skills introduced during the course, which have been positively rated by over 90% of participants from 2007 to 2018.

### Building multidisciplinary teams

The course brings together multidisciplinary teams including investigators, data managers, project coordinators, public health practitioners, administrators, and institutional authorities from different sectors concerned with research for health (Table 2). Participants are engaged on a level playing field of mutual interest and respect to plan their projects together. Constructive feedback is encouraged and normalized, providing opportunities for individuals approaching the research project from different perspectives to collaborate, learn from one another, and contribute to each other’s success.

**Table 2:** Regional participants in “Planning for Success” training conducted by the LAC-RTC and network institutions.

Country	Total	Male	Female	% Participants by Sector	
Bolivia	20	7	13	100	Academia
Colombia	271	106	165	33	Academia
				8	Government
				8	Healthcare delivery
				51	Research institution
Costa Rica	6	3	3	50	Academia
				50	Research institution
Cuba	3	0	3	100	Research institution
Ecuador	30	10	20	100	Academia
El Salvador	3	1	2	100	Government
Grenada	14	5	9	64	Government
				36	Academia
Guatemala	9	3	6	100	Academia
Guyana	34	14	20	15	Academia
				32	Government
				15	Healthcare delivery
				26	PAHO
				3	Research institution
				9	Unknown
Honduras	40	10	30	85	Academia
				15	Government
Jamaica	21	7	14	95	Academia
				5	Healthcare delivery
Peru	15	14	1	100	Academia
Dominican Republic	1	0	1	100	Academia
Suriname	44	13	31	16	Academia
				50	Government
				34	Healthcare delivery
Total	511	193	318		

**Abbreviations**: LAC, Latin American and Caribbean; PAHO,Pan American Health Organization; RTC, Regional Training Center.

## Dissemination: An institutional focus

Building health research capacity, particularly to address NTDs, requires a critical mass of competent and coordinated research teams. Development of skills in project planning and evaluation connects individual and institutional capacities, empowering research teams. Since CIDEIM’s introduction to the “Planning for Success” initiative in 2007, 511 individuals, including 85 trainers, in 30 institutions in Latin America and the Caribbean have been trained in “Planning for Success” methodology (Table 2). Overall participants have been affiliated with academia (47%), research institutions (29%), government (14%), healthcare delivery (8%), Pan-American Health Organization (PAHO) (2%), and undefined (1%). RTC partner institutions have increased and continue to further increase these numbers through replication of training within and beyond their institutions. This ongoing regional experience illustrates key characteristics of the dissemination strategy and the institutional appropriation that have sustained the “Planning for Success” initiative.

### TTTs: Starting point for standardized, self-replicating dissemination

During piloting of the EPPE Skill-Building course (2002–2003), MIM grantees ratified the value of the training and expressed the desire to train their team members and others. In response, TDR developed the TTT course to facilitate dissemination [10]. The TTT course provides mentored experience and practical guidance to support the presentation and uptake of the project management content. Facilitation, group management, training skills, and logistical considerations for organizing the Skill-Building course are also addressed. Additionally, a system for consistently identifying and preparing new trainers was established. Future trainers are identified during the Skill-Building course, considering innate teaching capacities and motivation to multiply project management skills. Candidates are invited to participate in a TTT course. Training materials for Skill-Building and TTT courses are available in English, Spanish, Portuguese, and French (http://www.who.int/tdr/publications/topics/project_planning_training/en/). These standardized materials and system of replication have facilitated propagation in person and through a virtual course (http://www.cideim.org.co/moodle/) while minimizing the risk for inconsistent course quality and divergence from core messages.

### Dissemination: A pay-it-forward approach

**Fig 3:**
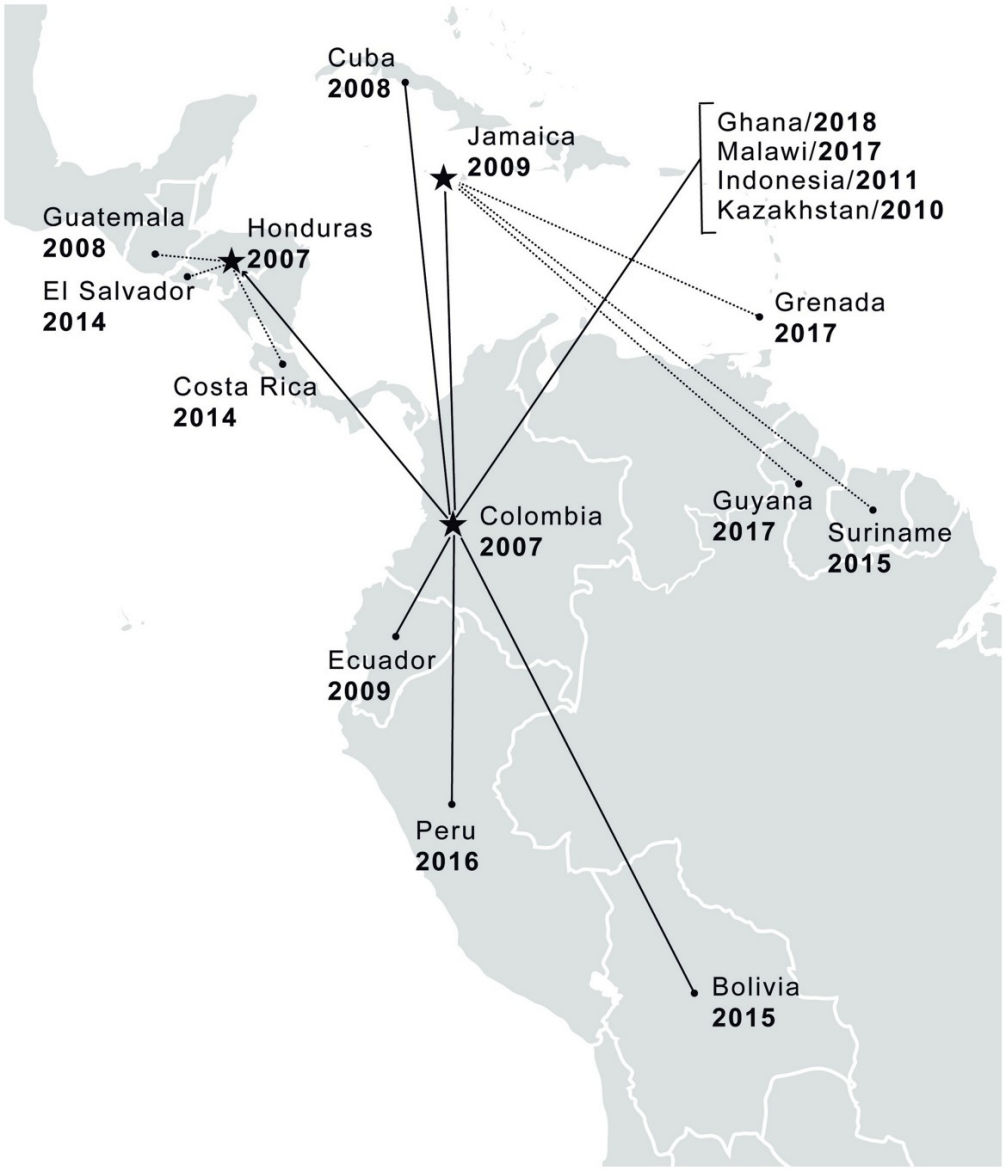
Geographic outreach of “Planning for Success” training: Countries and year of introduction of EPPE by the LAC-RTC and network institutions. Stars represent the RTC for the Americas (CIDEIM in Colombia) and network institutions that have become training hubs. Filled circles indicate countries where network institutions have replicated the EPPE course in their institution. Solid lines indicate the transfer of training directly imparted by CIDEIM. Dotted lines illustrate the secondary cascade of training by other network institutions with support and accompaniment by the RTC. (Illustration created from wikipedia/commons/0/03/BlankMap-World6.svg open-source map adapted using Adobe Illustrator from the CS bundle.) CIDEIM, Centro Internacional de Entrenamiento e Investigaciones Médicas; EPPE, Effective Project Planning and Evaluation; LAC, Latin American and Caribbean; RTC, Regional Training Center.

A commitment to a bottom-up leadership and distributed empowerment approach, together with the mutual leveraging of the institutional resources and capacities of CIDEIM, TDR, PAHO, and national partners, has catalyzed the dissemination of the “Planning for Success” initiative and scalable and sustainable research capacity building. CIDEIM initially built upon its established national research partnerships to develop training experience and increase its trainer pool. With the support of PAHO and TDR, CIDEIM expanded EPPE training to the Andean region, Central America, and Jamaica in the Caribbean (Fig 3). Importantly, several network institutions have also invested in the institutionalization of “Planning for Success” and participated in the dissemination of this initiative in the region. Paying it forward occurs when Skill-Building courses are conducted in regional institutions with the intent and understanding that those introduced to this training will join the regional network in building project management skills among health research teams by replicating and disseminating the training to others needing these skills. Exemplifying this “cascade” process, the Autonomous University of Honduras has become a training hub in Central America disseminating EPPE capacity to Guatemala, El Salvador, and Costa Rica. Similarly, the Caribbean Institute for Health Research (CAIHR) has served as the epicenter for conducting training in Guyana, Suriname, and Grenada. Furthermore, in addition to replicating the short training course for investigators and faculty through continuing education, several network institutions have integrated the principles and methods into their undergraduate or postgraduate programs, assuring the sustainability of the benefits among future generations (Table 1).

## Beyond project management: Building upon “Planning for Success”

Beyond the Americas, CIDEIM has also introduced “Planning for Success” training capacity to RTCs in Indonesia, Kazakhstan, and Ghana and to the University of Malawi, promoting the parallel dissemination of project management skills in Central and South East Asia and Western and Eastern Africa. Through this process of inter- and intraregional dissemination, the project management course has been adapted to address a broad range of health research needs, to incorporate other project planning and monitoring challenges such as budgeting, and to allow integration into university curricula, thereby responding to participant evaluations and trainer experiences [14]. Moreover, the “Planning for Success” experience has served as a gateway to the development and dissemination of other capacity-building courses and training opportunities such as the Good Health Research Practices course sponsored by TDR, with the goal of applying good clinical practice concepts to health research involving human participation beyond clinical trials [15].

The beneficial impact and intermediate outcomes of the “Planning for Success” approach to building project management capacity among health research teams are substantiated by its sustained dissemination and appropriation and the incorporation of the methodology into institutional practices and curricula by the RTC and network institutions. The training experience and methodology described in this Viewpoint deliver a valued product to participants, namely a clear, feasible plan for their projects, thereby promoting institutional appropriation of research skill building through active learning and outcome-oriented courses that complement and are readily integrated into academic curricula. The institutional focus, regional leadership and synergistic partnerships established through the “Planning for Success” initiative have provided a foundation for strengthening regional health research.

**Key points**

- Effective and sustainable research teams need to have the skills to plan the preparation, conduct, and delivery of research (i.e., project planning, monitoring, and evaluation of research activities), especially to address neglected tropical diseases (NTDs) in resource-constrained low- and middle-income countries (LMICs). These skills are seldom included in the curricula of higher education.
- Strategic partnerships among institutions in LMICs, UNICEF/UNDP/World Bank/WHO Special Programme for Research and Training in Tropical Diseases (TDR), and WHO regional offices have supported the development of human capital by introducing and disseminating project planning and evaluation competencies using a propagable Train-the-Trainer approach. This initiative has contributed to advancing WHO’s Strategy on Research for Health and developing regional collaborative networks.
- A regional network of experts and a cadre of over 500 professionals with skills in “Planning for Success” in research for health, including 85 trainers in 30 different institutions in Latin America and the Caribbean, have been trained in the methodology. The institutional focus, regional leadership, and synergistic partnerships have provided a foundation for strengthening regional health research capacity.
